# Biomechanical Evaluation of a Low-Invasive Elbow Medial Collateral Ligament Reconstruction Technique With Fascia and Tendon Patches

**DOI:** 10.3389/fbioe.2022.831545

**Published:** 2022-03-22

**Authors:** Wenjun Liu, Hao Xiong, Shuai Chen, Jingwei Zhang, Wei Wang, Yun Qian, Cunyi Fan

**Affiliations:** ^1^ Department of Orthopedics, Shanghai Jiao Tong University Affiliated Sixth People’s Hospital, Shanghai, China; ^2^ Shanghai Engineering Research Center for Orthopaedic Material Innovation and Tissue Regeneration, Shanghai, China; ^3^ Department of Orthopedics, Shanghai Jiao Tong University Affiliated Sixth People’s Hospital South Campus, Shanghai, China

**Keywords:** elbow, MCL reconstruction, biomechanics, elbow valgus stability, fascia patch, tendon patch

## Abstract

**Background:** Injury to the medial collateral ligament (MCL) of the elbow has become increasingly common in sports, and the elbow is prone to contracture and ossification in trauma. Effective reconstruction of the MCL with reduction of irritation to the elbow has rarely been reported. This study introduces a minimally invasive elbow MCL reconstruction technique and evaluates the valgus stability.

**Methods:** Eight fresh-frozen elbow specimens underwent reconstruction of the anterior bundle of the MCL (AMCL) first with the flexor carpi ulnaris fascia patches, followed by reconstruction of the posterior bundle of the MCL (PMCL) with the triceps tendon patches. The valgus angles of each specimen were examined in three stages as follows: intact MCL, reconstruction of the AMCL alone, and reconstruction of the MCL (including AMCL and PMCL). Finally, specimens were loaded to failure, and failure modes were recorded.

**Results:** AMCL reconstruction alone had similar valgus stability at all testing angles (*p* = 0.080, 30° flexion; *p* = 0.064, 60° flexion; *p* = 0.151, 90° flexion; *p* = 0.283, 120° flexion) compared with the intact MCL, as did MCL reconstruction (*p* = 0.951, 30° flexion; *p* = 0.739, 60° flexion; *p* = 0.841, 90° flexion; *p* = 0.538, 120° flexion). More importantly, a significant difference existed between the MCL reconstruction and the AMCL reconstruction alone at 30° flexion (*p* = 0.043) and 60° flexion (*p* = 0.013) but not at the 90° flexion (*p* = 0.369) and 120° flexion (*p* = 0.879). The mean maximum failure torque of MCL reconstruction was 24.02 Nm at 90° elbow flexion.

**Conclusion:** Both AMCL reconstruction alone and MCL reconstruction provided valgus stability comparable with the native MCL, and importantly, MCL reconstruction provided more valgus stability than AMCL reconstruction alone at 30° flexion and 60° flexion of the elbow. Therefore, the new MCL reconstruction technique might be a useful guide for the treatment of elbow MCL injuries or deficiencies.

## Introduction

In sports injuries, damage to the medial collateral ligament (MCL) of the elbow has become increasingly common (Petty et al., 2004). In elbow trauma, the elbow soft tissue develops posttraumatic contracture in 50% of patients (Anakwe et al., 2011) who experience elbow dislocation or fracture; furthermore, the elbow is prone to heterotopic ossification (HO) in 37% of patients who undergo open reduction and internal fixation (Foruria et al., 2013). The elbow is sensitive to surgical trauma, which is a risk factor for stiffness progression (Qian et al., 2020). The tendon grafts used in reconstruction techniques were confirmed to have undergone ossification (Wear et al., 2011; Kodde et al., 2012; Andrachuk et al., 2016) and occurred in the early stages of ligament reconstruction (Park et al., 2018). Considering that the elbow is sensitive to trauma, it is a severe challenge to reduce irritation to the elbow and effectively reconstruct the MCL.

Previous studies demonstrated that the anterior bundle of the MCL (AMCL) was the primary restraint to valgus stress (Morrey and An, 1983; Hassan et al., 2015; Frangiamore et al., 2017), the posterior bundle of the MCL (PMCL) could resist motion of the humero-ulnar joint (Shukla et al., 2016), and the transverse bundle of the MCL had little effect on maintaining medial stability (Morrey and An, 1983). Based on the importance of AMCL, many alternative reconstruction techniques for the role of AMCL had been proposed (Rohrbough et al., 2002; Paletta and Wright, 2006; Schwartz et al., 2008; Cain et al., 2010). The reconstruction techniques, using tendon grafts and bulky bone tunnels, showed good results in stabilizing the medial elbow (Rohrbough et al., 2002; Paletta and Wright, 2006; Schwartz et al., 2008; Cain et al., 2010), but it exposed the elbow to further trauma, causing a 5% odds of HO after MCL reconstruction (Park et al., 2018). Simultaneously, traditional reconstruction techniques demanded additional incisions for obtaining tendon grafts that might cause dysfunction in the tendon supply area (Vitale and Ahmad, 2008; Cain et al., 2010). Therefore, a less invasive ligament reconstruction technique is urgently needed for those patients who are prone to contracture or ossification.

With advancements in research on AMCL, some authors reported that the proximal and distal parts of the insertion of the AMCL played different roles in maintaining medial elbow stability (Hassan et al., 2015; Frangiamore et al., 2017). In addition, some reports claimed that the PMCL played a vital part in maintaining the posteromedial stability of the elbow and suggested a reconstruction of the PMCL after injury (Shukla et al., 2016; Shukla et al., 2018).

Considering the complication rate of elbow surgery and the role of AMCL and PMCL, we performed the fan shape reconstruction technique, which was minimally invasive to the bone. In this study, we introduce the new MCL reconstruction technique and evaluate the valgus stability with biomechanical analyses. We hypothesized that this technique could provide the same valgus stability as intact ligaments.

## Methods

### Preparation of Specimens

Eight fresh-frozen human cadaveric upper extremities (three women and five men; donated for medical research to a tissue bank and purchased by our institution) were used for this study. The mean age of the cadavers was 61.1 years (age range: 52–67 years). The specimens were stored at −20°C and thawed at room temperature for 24 h. We preserved the hand, forearm, and distal half of the upper arm, including the skin and muscles.

### Reconstruction Techniques

**FIGURE 1:**
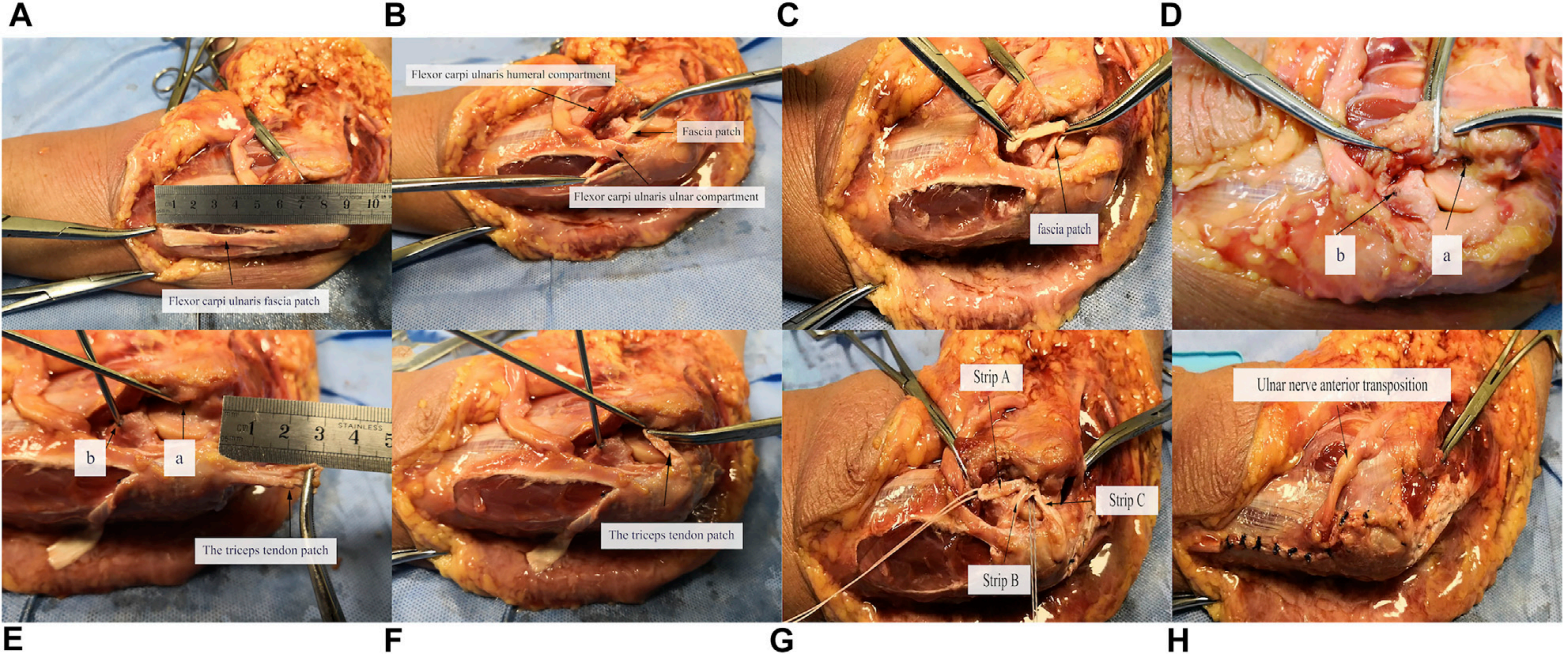
Illustration of technical procedures. **(A)** Preparation of fascia patch; **(B,C)** course of fascia patch; **(D)** option of reconstruction location, a: origin point of AMCL, b: center of ulnar attachments of AMCL on ulnar ridge; **(E)** creation of bone tunnels and preparation of triceps tendon patch; **(F)** course of triceps tendon patch; **(G)** fixation of fascia and tendon patches; **(H)** suture of remaining fascia patch and common flexor tendon, anterior transposition of ulnar nerve.

Using a medial incision, we carefully split the flexor-pronator muscle mass without damaging the flexor carpi ulnaris fascia patch and exposed the origins and insertions of the MCL (Supplementary Video S1). The flexor carpi ulnaris fascia patch was released from three sides of the muscle surface, whereas the proximal end remained intact 2 cm distal to the joint line (Figure 1A). The size of the fascia patch was approximately 6 cm long, 1 cm wide, and 2 mm thick (Figure 1A). The triceps tendon patch was released on three sides with the distal end at the insertion (Figure 1E). The size of the tendon patch was approximately 3 cm long, 0.5 cm wide, and 2 mm thick (Figure 1E). To prevent the patches from being further torn, the patches were sutured to their terminals by absorbable sutures.

The MCL reconstruction was performed at 90° of elbow flexion. At the stage of reconstruction of the AMCL alone, the prepared fascia patch was placed beneath the flexor carpi ulnaris muscle, close to the posteromedial part of the sublime tubercle, and extended proximally to the medial epicondyle (Figure 1B). The fascial patch was tensioned toward the medial epicondyle to determine the appropriate length and then fixed at the origin of the AMCL using a suture anchor (Twinfix; Smith & Nephew, Andover, MA, USA). Then, the patch was reversed and tensioned toward the ulnar ridge (Figure 1C). After obtaining the proper length, the fascia patch was fixed on the ulnar ridge with a suture anchor, distal to the sublime tubercle, and approximately 7 mm distal to the joint line, at the center of ulnar attachments of the AMCL (Figure 1D) (Frangiamore et al., 2018). A 2.5-mm K-wire was used to create suture anchor tunnels in the sublime tubercle and the medial epicondyle (Figure 1E). These drill holes did not broach the distal bone cortex. At the stage of reconstruction of the PMCL (Figure 1F), the triceps tendon patch was tensioned toward the medial epicondyle and fixed at the same point with the fascial patch on the medial epicondyle after determining the appropriate length of the tendon patch. The MCL reconstruction with strips A, B, and C resembles a fan shape (Figure 1G), named the Fan technique. After MCL reconstruction, the ulnar nerve was transposed subcutaneously and anteriorly, and then, the remaining flexor carpi ulnaris fascia patch and the common flexor tendon were sutured separately with interrupted sutures (Figure 1H).

### Biomechanical Test

**FIGURE 2:**
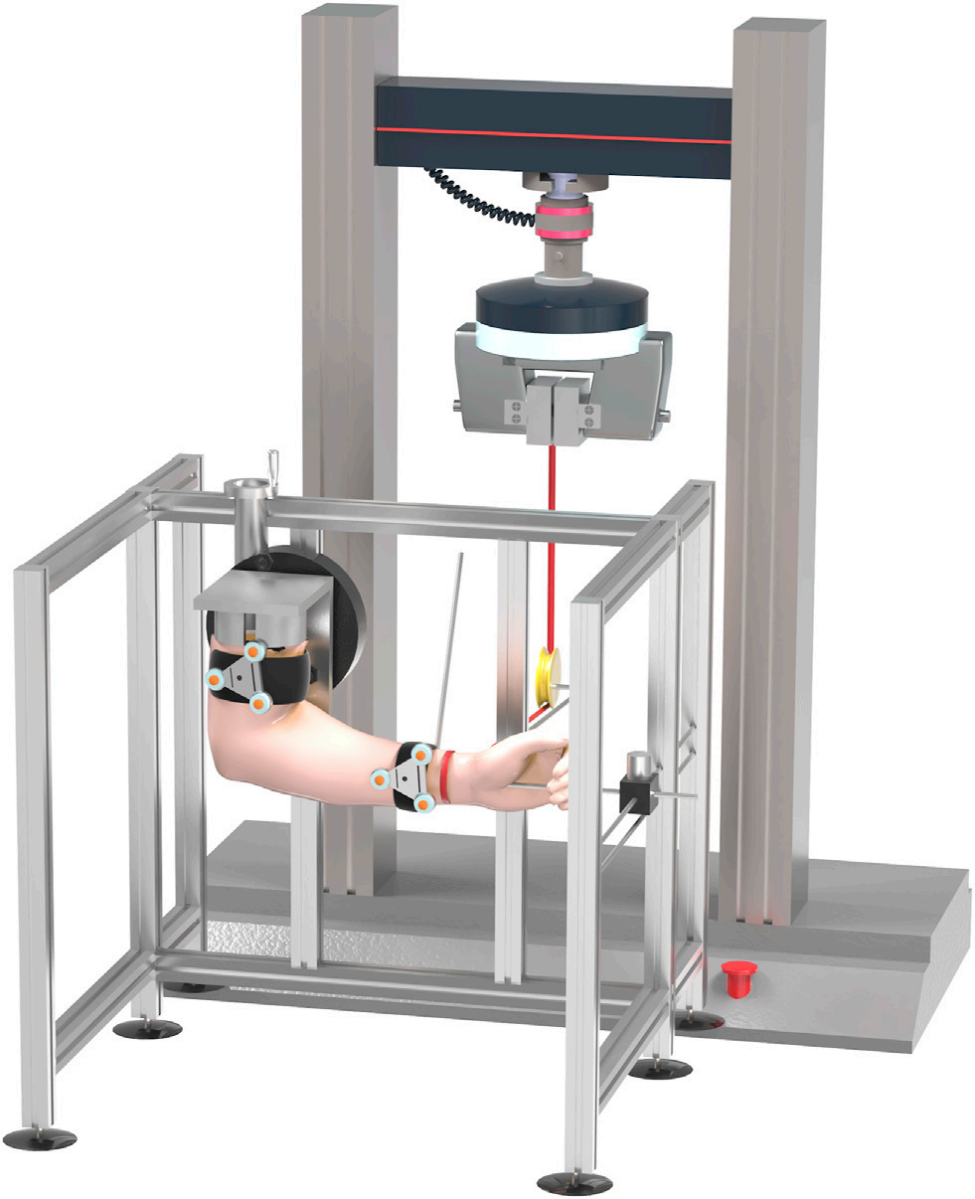
Schematic illustration of testing system. This image shows test under valgus stress with 2 Nm of torque, whereas elbow is in 90° of flexion, and forearm is in neutral.

The specimens were tested on the Instron 5569 Materials Testing System (Instron Corp, Canton, MA, USA). The proximal end of the specimen was potted in a custom fixture (Figure 2, Supplementary Figures S1, S2) using denture base acrylic resins. The distal end was fixed in the slider, which could not rotate to ensure that the forearm was in a neutral position and could only slide horizontally. One end of the 0.2-mm flexible steel wire rope was placed on the ulna, 20 cm from the elbow joint activity center, wherein loading this location created an elbow valgus moment. The other end was fixed in a small module, which could be clamped by the MTS clamps. By adjusting the pulley position, the wire rope could be first passed horizontally through the bottom of the pulley and then vertically upward linked to the MTS system. A preload of 2 Nm was applied, followed by 10 cycles, and held a final 2 Nm load for 60 s (Dugas et al., 2016). Kinematics was defined as the joint coordinate system of ulnar motion relative to the humerus, as recommended by the International Society of Biomechanics (Wu et al., 2005). The valgus angle was measured by the three-dimensional motion-capture system (Optotrak Certus motion-capture system; Northern Digital Inc., Waterloo, ON, Canada) at a torque of 2 Nm. One smart marker set was attached to the forearm and the other to the humerus. One smart marker set contained three markers, which consisted of infrared diodes. The marker set acted as a single “rigid body,” allowing it to specify its position in space. The markers allowed determining the relative displacement of the two rigid bodies (the forearm and humerus). The three-dimensional motion-capture system could calculate the coordinates of the markers, the centroid coordinates, and orientation angles. The MATLAB software (version R2016; Math Works, Natick, MA) was used to analyze the elbow valgus angle from the motion trajectory.

Each specimen was operated on in three different stages (Table 1). After stage A, the AMCL and PMCL were transected, then stages B and C were performed. After all testing, the specimen was positioned at 90° of flexion and loaded to failure at a rate of 0.2 mm/s. Failure was defined as a sudden decrease in tension with increasing elbow valgus. The maximum tension and the failure mode of MCL reconstruction were recorded.

**TABLE 1:** Stages of medial collateral ligament reconstruction.

Stage	Ligaments status
Stage A	Intact MCL
Stage B	Reconstruction of AMCL alone
Stage C	Reconstruction of MCL

### Statistical Analysis

Statistical data were analyzed using SPSS (IBM Corporation Ltd., Armonk, NY, USA). Each variable was reported as the actual measured value or the mean ± standard deviation (95% confidence intervals). After verification of the normal distribution, Levene's test was used to assess the equality of variance. Values were compared by use of one-way analysis of variance. Last, Tukey's *post-hoc* test was used to adjust for multiple comparisons with a significance criterion of *p* < 0.05.

## Results

**FIGURE 3:**
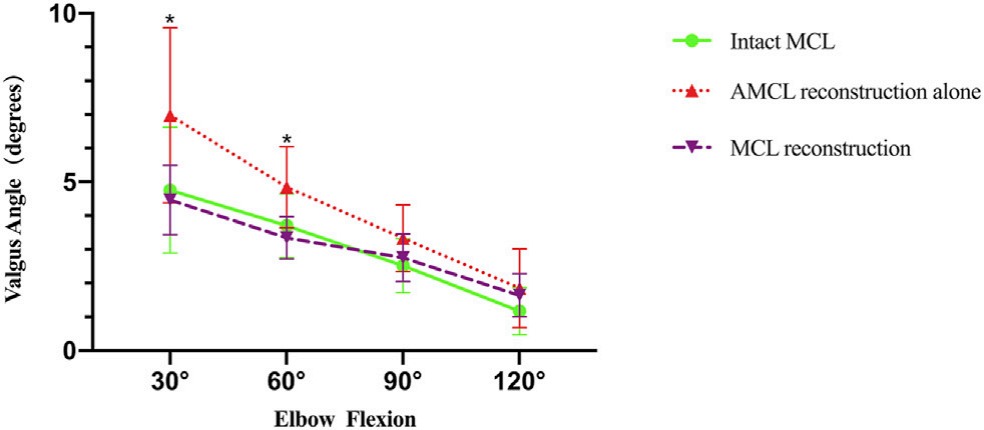
Line graph showing valgus angle applied 2 Nm torque at different elbow flexion angles for intact MCL, AMCL reconstruction alone, MCL reconstruction. Error bars indicate standard deviation. *p < 0.05.

A significant difference existed among the intact MCL, AMCL reconstruction alone, and the MCL reconstruction at 30° (*p* = 0.033) and 60° flexion (*p* = 0.013) but not at 90° (*p* = 0.160) and 120° flexion (*p* = 0.298) (Table 2). Intergroup comparison showed that reconstructing AMCL alone had a similar contribution to elbow medial stability at all testing angles (*p* = 0.080, 30° flexion; *p* = 0.064, 60° flexion; *p* = 0.151, 90° flexion; *p* = 0.283, 120° flexion) as the intact MCL. Reconstructing MCL had the ability to maintain elbow medial stability at all angles of testing (*p* = 0.951, 30° flexion; *p* = 0.739, 60° flexion; *p* = 0.841, 90° flexion; *p* = 0.538, 120° flexion) similar to intact MCL. More importantly, a significant difference existed between the MCL reconstruction and reconstruction of the AMCL alone at 30° flexion (*p* = 0.043) and 60° flexion (*p* = 0.013), although not at 90° flexion (*p* = 0.369) and 120° flexion (*p* = 0.879). In the line chart (Figure 3), at 30° and 60° flexion, the MCL reconstruction had an advantage over the AMCL alone with respect to resisting valgus stress, implying that reconstructed MCL contributed significantly more to maintaining medial elbow stability than reconstructed AMCL alone.

**TABLE 2:** Valgus angle and statistical difference.

Elbow flexion	Valgus angle^°^ ^a^			Levene	*p*-value			
	Intact MCL(A)	Recon-AMCL alone(B)	Recon-MCL(C)		ANOVA	TUKEY		
						A *vs*. B	A *vs*. C	B *vs*. C
30°	4.76 ± 1.86 (3.20–6.32)	6.98 ± 2.60 (4.80–9.15)	4.47 ± 1.03 (3.61–5.32)	0.112	0.033	0.080	0.951	0.043
60°	3.70 ± 0.95 (2.91–4.49)	4.85 ± 1.20 (3.84–5.85)	3.35 ± 0.63 (2.82–3.87)	0.077	0.013	0.064	0.739	0.013
90°	2.52 ± 0.80 (1.85–3.19)	3.34 ± 0.99 (2.51–4.16)	2.76 ± 0.70 (2.17–3.35)	0.322	0.160	0.151	0.841	0.369
120°	1.17 ± 0.70 (0.59–1.75)	1.85 ± 1.17 (0.87–2.83)	1.64 ± 0.64 (1.11–2.17)	0.110	0.298	0.283	0.538	0.879

a Values are given as mean ± standard deviation (95% confidence interval). Results of Levene test showed that data were consistent with equality of variance. A *vs*. B: reconstruction AMCL, alone compared with intact MCL; A *vs*. C, reconstruction MCL, compared with intact MCL; B *vs*. C, reconstruction AMCL, alone compared with reconstruction MCL. A significance criterion of *p* < 0.05. ANOVA, analysis of variance

The mean maximum failure torque of MCL (including AMCL and PMCL) reconstruction at 90° flexion was 24.02 Nm (Table 3). The modes of failure were strip A tear (3/8) and strip B tear (5/8) (Table 3).

**TABLE 3:** Maximum valgus failure test.

Failure test	Cadaver no.								
	1	2	3	4	5	6	7	8	Mean
MVF^a^	140.5	132.8	123.7	113.9	101.9	109.4	117.4	121.1	120.09
MVT^b^	28.1	26.56	24.74	22.78	20.38	21.88	23.48	24.22	24.02
Mode^c^	SBT	SBT	SAT	SAT	SBT	SBT	SAT	SBT	

a Values are given as maximum valgus force (MVF).

b Values are given as maximum valgus torque (MVT).

c Mode of maximum valgus failure. SAT, Strip A Tear; SBT, Strip B Tear.

## Discussion

Our AMCL reconstruction alone and MCL reconstruction techniques were sufficient to maintain elbow stability. Both AMCL reconstruction alone and MCL reconstruction provided valgus stability comparable with the native MCL at 30°, 60°, 90°, and 120° of elbow flexion. Moreover, MCL reconstruction was better than AMCL reconstruction alone in maintaining valgus stability at 30° flexion and 60° flexion. The good results of the maximum failure test for the MCL reconstruction further demonstrated the excellent ability of the new MCL reconstruction technique to maintain stability.

**FIGURE 4:**
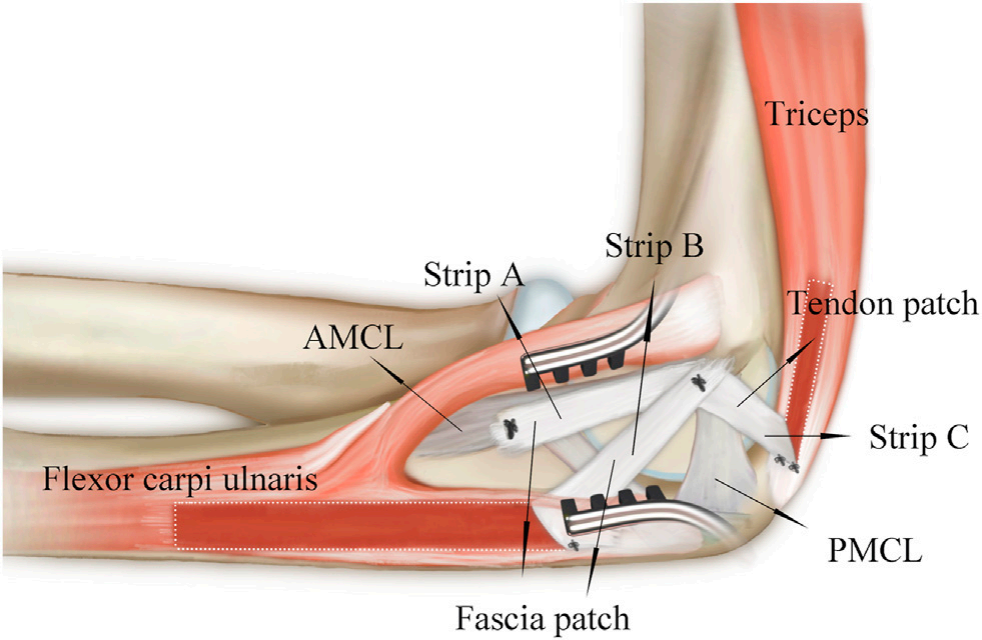
Schematic diagram shows position of reconstructed ligaments in relation to original ligaments.

In previous anatomical studies of AMCL, the insertion of AMCL was confirmed on the sublime tubercle of the ulnar ridge (Frangiamore et al., 2018; Farrow et al., 2011). Frangiamore et al. (2017) found that the proximal part of the insertion contributed more to stability at a higher elbow flexion angle, whereas the distal part of the insertion played a greater role at lower flexion angles. Hassan et al. (2015) suggested that the proximal half of the insertion footprint had a primary role in maintaining posteromedial stability of the elbow, whereas the distal half of the insertion tear had no significance in the biomechanical study. Considering the importance of the proximal ligament insertion, we proposed the reconstruction of AMCL with strips A and B to simulate the function of native AMCL (Figure 1G). Strip A was proximal to the distal half of the insertion and could mimic the role of the proximal half of the AMCL insertion (Figure 4). Strip B was proximal to the proximal half of the insertion, strengthening the role of the proximal half of the AMCL insertion. The Jobe technique (Mullen et al., 2002; Armstrong et al., 2005; Paletta et al., 2006) and the Docking technique (Armstrong et al., 2005; Paletta et al., 2006; Ciccotti et al., 2009) used double-strand reconstruction such that the two strands of the palmaris graft converged into a single-strand at the epicondyle. The direction of strip A was similar to the lateral strand of the two strands in the Jobe and Docking techniques. The direction of strip B was similar to but posterior to the medial strand. This posterior location was close to the functional area of AMCL. Our new AMCL reconstruction technique had the characteristics of the reconstructed ligaments in the traditional technique and was closer to the functional area than the traditional technique.

Based on the role of PMCL in resisting motion of the humero-ulnar joint (Shukla et al., 2016), the new technique was also used to perform PMCL reconstruction. Baumfeld et al. (2010) found that the central and medial portions of the triceps tendon were significantly thicker and stiffer than the lateral portion. Therefore, we used the medial portion of the triceps tendon to reconstruct the PMCL. In the reconstruction of PMCL, the triceps tendon patch was posterior to native PMCL, which was 1.6 mm distal, 9.8 mm anterior to the olecranon tip, and 4.4 mm posterior to the joint line (Ciccotti et al., 2009). The position of the reconstructed PMCL was closer to the proximal end of the ulna than the original PMCL. Thus, the reconstructed PMCL might play a role in maintaining posteromedial stability.

In previous biomechanical studies (Mullen et al., 2002), the Jobe technique was effective at 30°, 60°, and 90° flexion and invalid at 120° flexion. Moreover, at 120° flexion, the strength of the reconstructed ligament was only 89% of the strength of the original ligaments. Paletta et al. (2006) discovered that the Jobe and Docking techniques did not reproduce the biomechanical profile of the native MCL. Bodendorfer et al. (2018) discovered that the Docking technique provided valgus stability to the medial elbow comparable with the native ligament only at 90° flexion. These reconstruction techniques could not truly achieve the ability of the original ligament to maintain elbow stability. Our results showed that both reconstructions of the AMCL alone and MCL achieved similar elbow stability as intact MCL. Reconstructing the MCL could provide more stability than reconstruction of the AMCL alone at 30° flexion and 60° flexion, but not at 90° flexion and 120° flexion, because in the latter two flexion angles, the gathering of muscles and docking of bony structures might limit the elbow valgus and cover up the ability of the strips. This result implied that reconstruction of MCL was an ideal technique when facing a valgus torque of more than 2.0 Nm at 30° flexion and 60° flexion.

The maximum failure torque of MCL reconstruction was 24.02 Nm at 90° flexion, where the valgus torque reached a peak in the throwing elbow (Ciccotti et al., 2009). The maximum torque of the natural ligament varied in different biomechanical studies. The maximum torque of the intact cadaver MCL was 18.8 Nm in the Palette study (Paletta et al., 2006), 22.7 Nm in the Hechtman study (Hechtman et al., 1998), 21.8 Nm in the Armstrong study (Armstrong et al., 2005), and 34 Nm in the Ahmad study (Ahmad et al., 2003). In our study, we did not test the maximum valgus failure of the native MCL. By comparison, it could be seen that our new reconstructed MCL was better than the intact ligament in several studies (Hechtman et al., 1998; Armstrong et al., 2005; Paletta et al., 2006) but worse than the intact ligament in one study (Ahmad et al., 2003). In the maximum failure experiment, Ahmad used young (mean age, 43 years) male cadavers, which might account for the large values of intact ligaments (Ahmad et al., 2003). The maximum failure torque of some previous reconstruction techniques did not reach the maximum failure level of intact ligaments. The maximum failure torque for the Jobe technique was 13.2 Nm (range, 8.0–22.7 Nm), for the Docking technique was 11.3 Nm (range, 8.2–14.3 Nm), and for the interference screw technique was 21.98 Nm (range, 13.4–30.55 Nm) (Watson et al., 2014). Many factors might contribute to differences in observations, such as the age of cadavers, bone quality, and surgical skills. In general, our new MCL reconstruction technique could achieve similar results as intact MCL; moreover, it seems better than other techniques in maximum failure experiments.

In previous techniques (Mullen et al., 2002; Armstrong et al., 2005; Paletta et al., 2006), a 3.5-mm K-wire was used to create a bone tunnel on the ulnar and medial epicondyle, and the distal bone cortex was disrupted. The bone loss led to tunnel fracture (Rohrbough et al., 2002; Paletta et al., 2006; Paletta and Wright, 2006; Schwartz et al., 2008; Cain et al., 2010) and HO (Wear et al., 2011; Kodde et al., 2012; Watson et al., 2014; Andrachuk et al., 2016; Park et al., 2018). The bone cortex in the narrow ligament function area was destroyed more in the traditional technique than in the new technique. The bone cortex in the narrow ligament function area was less destroyed in the new technique, which used a 2.5-mm K-wire without disrupting the distal bone cortex. Compared with previous techniques using tendon palmaris longus or hamstring tendons, this fan shape reconstruction technique using the fascia and tendon patches around the elbow had the potential to reduce damage and complications in the tendon supply area.

Our study has some limitations. The advanced age of the cadavers and their decreased bone density may have had a deleterious effect on the strength of anchor-bone fixation. Furthermore, the specimens' advanced age and frozen preservation could have affected the toughness and strength of the flexor carpi ulnaris fascia and triceps tendon, which may not accurately reflect the situation in young patients and achieve actual clinical outcomes. A limited sample may increase variability and lack statistical significance between the intact and reconstructed groups. Another limitation was that we did not test the tension and strength of the fascia and tendon. In addition, failure testing of MCL reconstruction cannot be repeated, which may increase variability. In future work, prospective studies are needed to analyze further the clinical value of this novel, minimally invasive reconstruction technique.

## Conclusion

This cadaveric study proved that both AMCL reconstruction alone and MCL reconstruction provided valgus stability comparable with the native MCL, and more importantly, MCL reconstruction provided more valgus stability than AMCL reconstruction alone at 30° flexion and 60° flexion of the elbow. Therefore, the new MCL reconstruction technique might be useful to guide the treatment of elbow MCL injuries or deficiencies.

## Data Availability

The original contributions presented in the study are included in the article/Supplementary Material, further inquiries can be directed to the corresponding authors.
