# Supplementary figures and images for: Biomechanical Evaluation of a Low-Invasive Elbow Medial Collateral Ligament Reconstruction Technique With Fascia and Tendon Patches
Source: Front Bioeng Biotechnol. 2022 Mar 22;10:831545. doi: 10.3389/fbioe.2022.831545 (PMC8980741; doi:10.3389/fbioe.2022.831545)

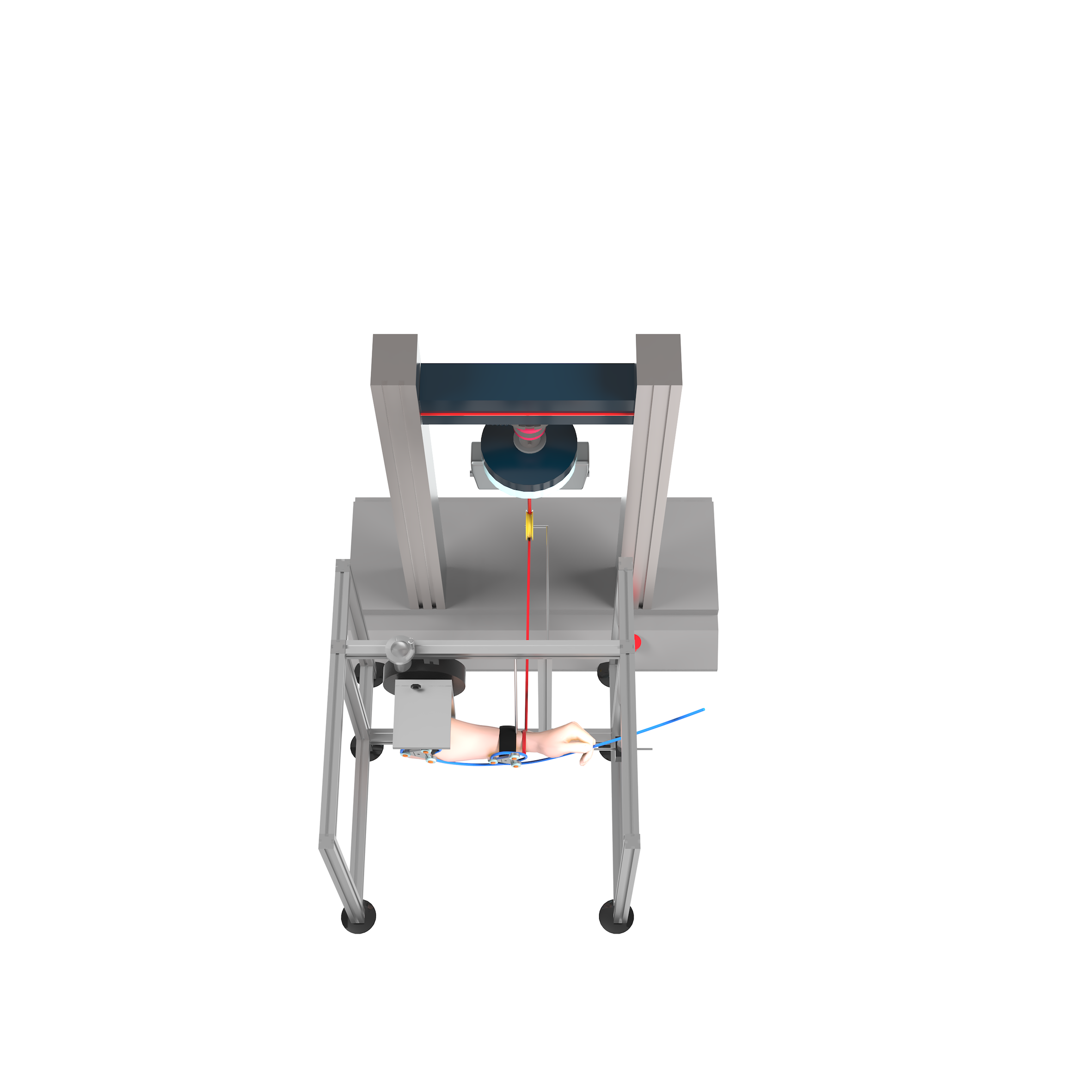

Supplement: Supplementary file 1 [file Image2.TIF]

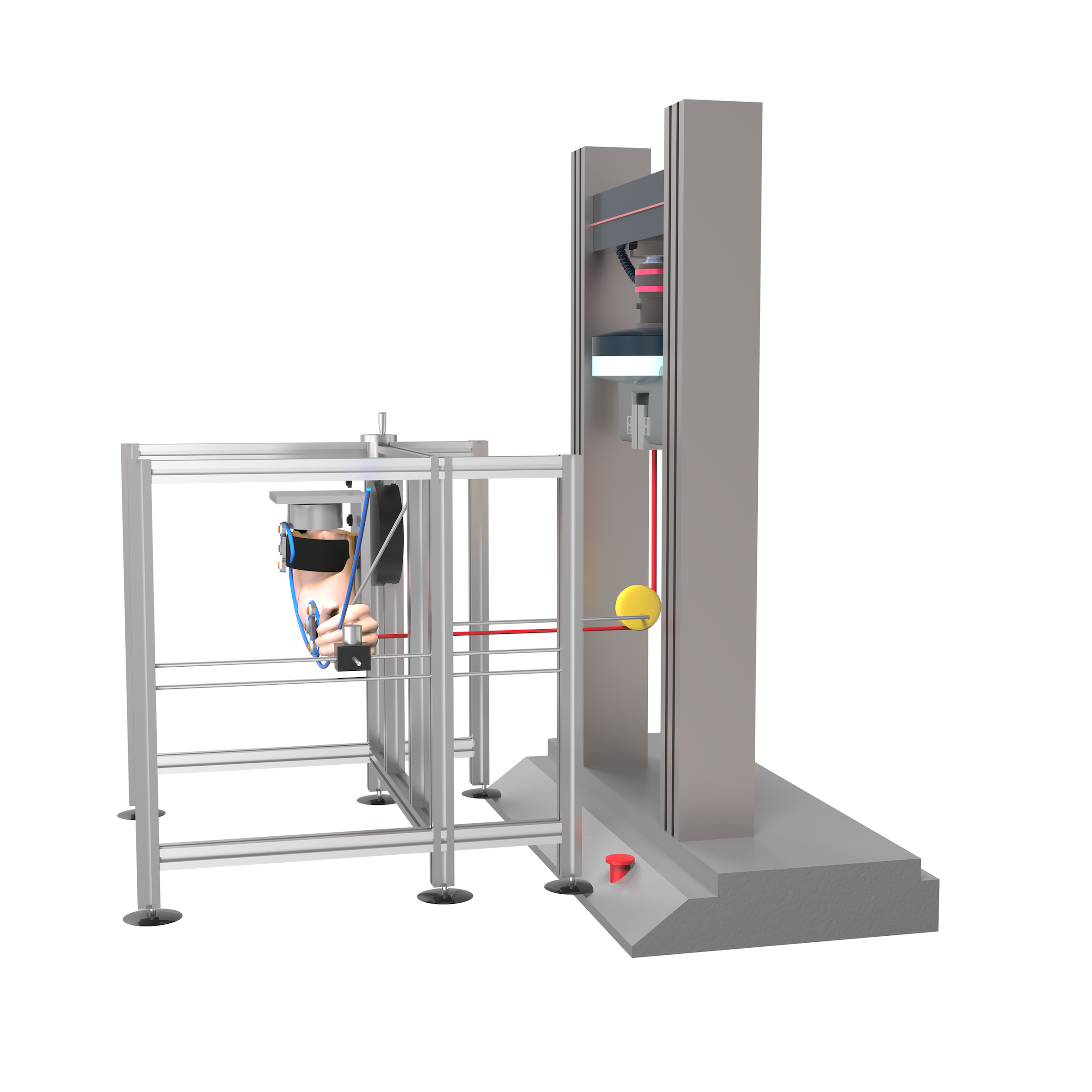

Supplement: Supplementary file 2 [file Image1.TIF]
